# A monoclinic polymorph of *N*-(3-chloro­phen­yl)benzamide

**DOI:** 10.1107/S1600536810040262

**Published:** 2010-10-13

**Authors:** Aamer Saeed, Muhammad Arshad, Jim Simpson

**Affiliations:** a Department of Chemistry, Quaid-i-Azam University, Islamabad 45320, Pakistan; b Chemistry Division, Directorate of Science, PINSTECH, Nilore, Islamabad, Pakistan; c Department of Chemistry, University of Otago, PO Box 56, Dunedin, New Zealand

## Abstract

The title compound, C_13_H_10_ClNO, (I), is a polymorph of the structure, (II), first reported by Gowda *et al.* [*Acta Cryst.* (2008), E**64**, o462]. In the original report, the compound crystallized in the ortho­rhom­bic space group *Pbca* (*Z* = 8), whereas the structure reported here is monoclinic *P*21/*c* (*Z* = 4). The principal difference between the two forms lies in the relative orientations of the phenyl and benzene rings [dihedral angle = 8.90 (13)° for (I) and 61.0 (1)° for (II)]. The inclination of the amide –CONH– units to the benzoyl ring is more similar [15.8 (7)° for (I) and 18.2 (2)° for (II)]. In both forms, the N—H bonds are *anti* to the 3-chloro substituents of the aniline rings. In the crystal, inter­molecular N—H⋯O hydrogen bonds form *C*(4) chains along *c*. These chains are bolstered by weak C—H⋯O inter­actions that generate *R*_2_^1^(6) and *R*_2_^1^(7) ring motifs.

## Related literature

For background to the biological activity of *N*-substituted benzamides and their use in synthesis, see: Saeed *et al.* (2010 ▶). For the ortho­rhom­bic polymorph of (I), see: Gowda, Tokarčík *et al.* (2008 ▶). For the structures of related chloro­phenyl­benzamides, see: Gowda *et al.* (2007*a* ▶,*b* ▶,*c* ▶); Gowda, Foro *et al.* (2008 ▶). For hydrogen-bond motifs, see: Bernstein *et al.* (1995 ▶). For bond-length data, see: Allen *et al.* (1987 ▶).
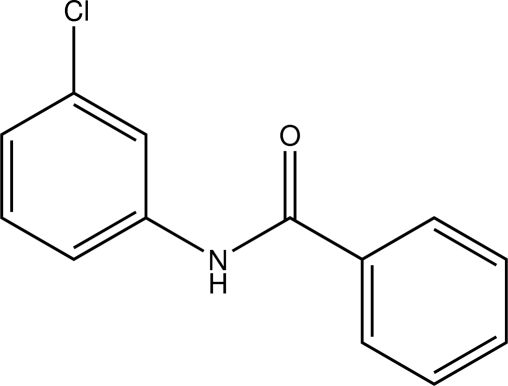


## Experimental

### 

#### Crystal data

C_13_H_10_ClNO
*M* *_r_* = 231.67
Monoclinic, 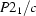

*a* = 12.5598 (17) Å
*b* = 10.2782 (14) Å
*c* = 9.0788 (13) Å
β = 109.421 (5)°
*V* = 1105.3 (3) Å^3^
*Z* = 4
Mo *K*α radiation
μ = 0.32 mm^−1^
*T* = 90 K
0.57 × 0.22 × 0.03 mm

#### Data collection

Bruker APEXII CCD diffractometer
Absorption correction: multi-scan (*SADABS*; Bruker, 2006 ▶) *T*_min_ = 0.743, *T*_max_ = 1.000
6385 measured reflections
2045 independent reflections
1475 reflections with *I* > 2σ(*I*)
*R*_int_ = 0.047

#### Refinement

*R*[*F* ^2^ > 2σ(*F* ^2^)] = 0.041
*wR*(*F* ^2^) = 0.104
*S* = 1.04
2045 reflections
148 parameters
H atoms treated by a mixture of independent and constrained refinement
Δρ_max_ = 0.29 e Å^−3^
Δρ_min_ = −0.26 e Å^−3^

### 

Data collection: *APEX2* (Bruker 2006 ▶); cell refinement: *APEX2* and *SAINT* (Bruker 2006 ▶); data reduction: *SAINT*; program(s) used to solve structure: *SHELXS97* (Sheldrick, 2008 ▶); program(s) used to refine structure: *SHELXL97* (Sheldrick, 2008 ▶) and *TITAN2000* (Hunter & Simpson, 1999 ▶); molecular graphics: *SHELXTL* (Sheldrick, 2008 ▶) and *Mercury* (Macrae *et al.*, 2008 ▶); software used to prepare material for publication: *SHELXL97*, *enCIFer* (Allen *et al.*, 2004 ▶), *PLATON* (Spek, 2009 ▶) and *publCIF* (Westrip, 2010 ▶).

## Supplementary Material

Crystal structure: contains datablocks global, I. DOI: 10.1107/S1600536810040262/lh5145sup1.cif

Structure factors: contains datablocks I. DOI: 10.1107/S1600536810040262/lh5145Isup2.hkl

Additional supplementary materials:  crystallographic information; 3D view; checkCIF report

## Figures and Tables

**Table 1:** Hydrogen-bond geometry (Å, °)

*D*—H⋯*A*	*D*—H	H⋯*A*	*D*⋯*A*	*D*—H⋯*A*
N1—H1⋯O1^i^	0.88 (2)	1.99 (2)	2.841 (2)	163 (2)
C7—H7⋯O1^i^	0.95	2.45	3.228 (3)	139
C13—H13⋯O1^i^	0.95	2.71	3.301 (3)	121

Symmetry code: (i) 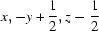.

**Fig. 1.**
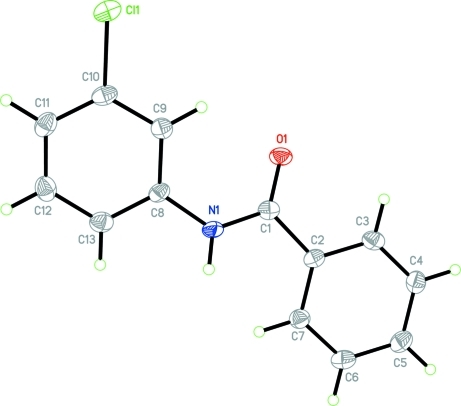
The structure of (I) with displacement ellipsoids for the non-hydrogen atoms drawn at the 50% probability level.

**Fig. 2.**
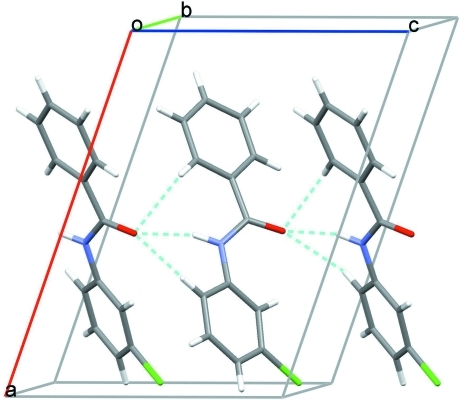
Crystal packing for (I) viewed along the b axis with hydrogen bonds drawn as dashed lines.

## supplementary crystallographic information

### Comment

N-substituted benzamides have numerous pharmaceutical and synthetic applications (Saeed *et al.*, 2010). The title compound, (I), is a monoclinic polymorph of the structure of this benzamide derivative which crystallizes in the space group P21/c. An alternative structure, (II), in the orthorhombic space group Pbca was reported previously by Gowda, Tokarčík *et al.*, (2008).

Bond distances in the molecule are normal (Allen *et al.*, 1987) and very similar to those in the orthorhombic polymorph and in closely related chlorophenylbenzamide derivatives (Gowda *et al.*, 2007*a*,*b*,*c*); Gowda, Foro *et al.*, 2008). However, the two polymorphs differ markedly in the relative orientations of the C2···C6 phenyl and C8···C13 benzene rings [dihedral angles 8.90 (13) for (I) and 61.0 (1) for (II)]. The inclination of the amide –C1O1N1H1- units to the C2···C6 ring is more similar [15.8 (7) for (I) and 18.2 (2) for (II). The N1–H1 bonds in both forms are *anti* to the Cl1 substituents of the C8···C13 aniline rings. This behaviour parallels that observed with *N*-(2-chlorophenyl)benzamide (Gowda *et al.*, 2007*a*) and *N*-(3,4-dichlorophenyl)benzamide (Gowda *et al.*, 2007*c*), whereas the a *syn* conformation is favoured in *N*-(2,3-dichlorophenyl)benzamide (Gowda *et al.*, 2007*b*)

In the crystal structure, Fig. 2, intermolecular N1–H1···O1 hydrogen bonds form C4 chains along the *c* axis (Bernstein *et al.* 1995). These chains are further stabilized by weak C7–H5···O1 and C13–H13···O1 interactions that generate an *R*_2_^1^(7) motif involving the an *ortho*-H atom from the phenyl ring and an *R*_2_^1^(6) motif incorporating an *ortho*-H atom from the chlorobenzene ring respectively.

### Experimental

Freshly distilled benzoyl chloride (1 mmol) in CHCl_3_ was treated with 3-chloroaniline (3.5 mmol) under a nitrogen atmosphere at reflux for 2.5 h. Upon cooling, the reaction mixture was diluted with CHCl_3_ and washed consecutively with 1 *M* aq HCl and saturated aq NaHCO_3_. The organic layer was dried over anhydrous sodium sulfate and concentrated under reduced pressure. Crystallization of the residue from ethanol afforded the title compound (83%) as colourless crystals: Anal. calcd. for C_13_H_10_Cl_N_O: C, 67.39; H, 4.35; N, 6.05; found: C, 67.21; H, 4.42; N, 6.10%.

### Refinement

The H atom bound to N1 was located in a difference map and refined isotropically. All other H-atoms were positioned geometrically and refined using a riding model with d(C—H) = 0.95 Å, *U*_iso_ = 1.2*U*_eq_ (C).

### Crystal data

**Table:**

C_13_H_10_ClNO	*F*(000) = 480
*M**_r_* = 231.67	*D*_x_ = 1.392 Mg m^−^^3^
Monoclinic, *P*2_1_/*c*	Mo *K*α radiation, λ = 0.71073 Å
Hall symbol: -P 2ybc	Cell parameters from 1106 reflections
*a* = 12.5598 (17) Å	θ = 2.6–25.3°
*b* = 10.2782 (14) Å	µ = 0.32 mm^−^^1^
*c* = 9.0788 (13) Å	*T* = 90 K
β = 109.421 (5)°	Rectangular plate, colourless
*V* = 1105.3 (3) Å^3^	0.57 × 0.22 × 0.03 mm
*Z* = 4	

### Data collection

**Table:**

Bruker APEXII CCD diffractometer	2045 independent reflections
Radiation source: fine-focus sealed tube	1475 reflections with *I* > 2σ(*I*)
graphite	*R*_int_ = 0.047
ω scans	θ_max_ = 25.5°, θ_min_ = 3.1°
Absorption correction: multi-scan (*SADABS*; Bruker, 2006)	*h* = −15→15
*T*_min_ = 0.743, *T*_max_ = 1.000	*k* = −12→12
6385 measured reflections	*l* = −9→10

### Refinement

**Table:**

Refinement on *F*^2^	Primary atom site location: structure-invariant direct methods
Least-squares matrix: full	Secondary atom site location: difference Fourier map
*R*[*F*^2^ > 2σ(*F*^2^)] = 0.041	Hydrogen site location: inferred from neighbouring sites
*wR*(*F*^2^) = 0.104	H atoms treated by a mixture of independent and constrained refinement
*S* = 1.04	*w* = 1/[σ^2^(*F*_o_^2^) + (0.0439*P*)^2^ + 0.2503*P*] where *P* = (*F*_o_^2^ + 2*F*_c_^2^)/3
2045 reflections	(Δ/σ)_max_ = 0.001
148 parameters	Δρ_max_ = 0.29 e Å^−^^3^
0 restraints	Δρ_min_ = −0.26 e Å^−^^3^

### Special details

**Table:**

Geometry. All e.s.d.'s (except the e.s.d. in the dihedral angle between two l.s. planes) are estimated using the full covariance matrix. The cell e.s.d.'s are taken into account individually in the estimation of e.s.d.'s in distances, angles and torsion angles; correlations between e.s.d.'s in cell parameters are only used when they are defined by crystal symmetry. An approximate (isotropic) treatment of cell e.s.d.'s is used for estimating e.s.d.'s involving l.s. planes.
Refinement. Refinement of *F*^2^ against ALL reflections. The weighted *R*-factor *wR* and goodness of fit *S* are based on *F*^2^, conventional *R*-factors *R* are based on *F*, with *F* set to zero for negative *F*^2^. The threshold expression of *F*^2^ > σ(*F*^2^) is used only for calculating *R*-factors(gt) *etc*. and is not relevant to the choice of reflections for refinement. *R*-factors based on *F*^2^ are statistically about twice as large as those based on *F*, and *R*- factors based on ALL data will be even larger.

### Fractional atomic coordinates and isotropic or equivalent isotropic displacement parameters (Å^2^)

**Table:**

	*x*	*y*	*z*	*U*_iso_*/*U*_eq_	
N1	0.58959 (16)	0.22008 (18)	0.5519 (2)	0.0208 (4)	
H1	0.5656 (19)	0.202 (2)	0.451 (3)	0.025*	
C1	0.52721 (18)	0.3046 (2)	0.6039 (3)	0.0187 (5)	
O1	0.55935 (12)	0.34896 (15)	0.73758 (17)	0.0223 (4)	
C2	0.41446 (18)	0.3409 (2)	0.4894 (2)	0.0180 (5)	
C3	0.35873 (19)	0.4469 (2)	0.5262 (3)	0.0217 (5)	
H3	0.3924	0.4929	0.6211	0.026*	
C4	0.25445 (19)	0.4852 (2)	0.4248 (3)	0.0233 (5)	
H4	0.2167	0.5572	0.4509	0.028*	
C5	0.20480 (19)	0.4194 (2)	0.2856 (3)	0.0251 (6)	
H5	0.1341	0.4473	0.2151	0.030*	
C7	0.36288 (18)	0.2734 (2)	0.3510 (3)	0.0215 (5)	
H7	0.3992	0.1998	0.3257	0.026*	
C8	0.69653 (18)	0.1670 (2)	0.6371 (2)	0.0188 (5)	
C9	0.77139 (19)	0.2275 (2)	0.7677 (3)	0.0204 (5)	
H9	0.7516	0.3062	0.8071	0.025*	
C10	0.87564 (19)	0.1698 (2)	0.8386 (3)	0.0220 (5)	
C11	0.9070 (2)	0.0546 (2)	0.7871 (3)	0.0242 (5)	
H11	0.9786	0.0165	0.8393	0.029*	
C12	0.8316 (2)	−0.0039 (2)	0.6576 (3)	0.0256 (6)	
H12	0.8516	−0.0832	0.6200	0.031*	
C13	0.72744 (19)	0.0511 (2)	0.5819 (3)	0.0232 (5)	
H13	0.6767	0.0101	0.4921	0.028*	
Cl1	0.96981 (5)	0.24773 (6)	1.00047 (7)	0.0317 (2)	
C6	0.25863 (19)	0.3132 (2)	0.2499 (3)	0.0259 (6)	
H6	0.2241	0.2669	0.1554	0.031*	

### Atomic displacement parameters (Å^2^)

**Table:**

	*U*^11^	*U*^22^	*U*^33^	*U*^12^	*U*^13^	*U*^23^
N1	0.0224 (10)	0.0271 (10)	0.0112 (10)	0.0031 (8)	0.0033 (9)	−0.0010 (9)
C1	0.0210 (12)	0.0203 (11)	0.0161 (12)	−0.0029 (9)	0.0079 (10)	0.0025 (10)
O1	0.0246 (9)	0.0287 (8)	0.0135 (9)	0.0002 (7)	0.0063 (7)	−0.0022 (7)
C2	0.0179 (11)	0.0220 (11)	0.0152 (12)	−0.0020 (9)	0.0067 (9)	0.0035 (9)
C3	0.0295 (13)	0.0221 (11)	0.0153 (12)	−0.0033 (10)	0.0100 (10)	0.0011 (10)
C4	0.0261 (13)	0.0247 (12)	0.0228 (13)	0.0053 (10)	0.0131 (11)	0.0038 (10)
C5	0.0184 (12)	0.0322 (13)	0.0237 (14)	0.0022 (10)	0.0056 (10)	0.0041 (11)
C7	0.0193 (12)	0.0248 (12)	0.0199 (12)	0.0003 (9)	0.0057 (10)	−0.0008 (10)
C8	0.0195 (12)	0.0229 (12)	0.0134 (12)	0.0015 (9)	0.0048 (10)	0.0052 (9)
C9	0.0252 (13)	0.0213 (12)	0.0161 (12)	0.0014 (9)	0.0085 (10)	0.0027 (10)
C10	0.0226 (12)	0.0285 (12)	0.0138 (12)	−0.0027 (10)	0.0043 (10)	0.0043 (10)
C11	0.0217 (12)	0.0309 (13)	0.0207 (13)	0.0052 (10)	0.0081 (10)	0.0081 (11)
C12	0.0329 (14)	0.0221 (12)	0.0244 (14)	0.0059 (10)	0.0129 (12)	0.0040 (10)
C13	0.0272 (13)	0.0240 (12)	0.0179 (12)	−0.0010 (10)	0.0068 (10)	0.0019 (10)
Cl1	0.0246 (3)	0.0376 (4)	0.0256 (4)	0.0006 (3)	−0.0013 (3)	−0.0033 (3)
C6	0.0227 (13)	0.0319 (13)	0.0204 (13)	−0.0038 (11)	0.0034 (10)	−0.0022 (11)

### Geometric parameters (Å, °)

**Table:**

N1—C1	1.356 (3)	C7—H7	0.9500
N1—C8	1.418 (3)	C8—C9	1.392 (3)
N1—H1	0.88 (2)	C8—C13	1.396 (3)
C1—O1	1.232 (2)	C9—C10	1.386 (3)
C1—C2	1.499 (3)	C9—H9	0.9500
C2—C7	1.392 (3)	C10—C11	1.378 (3)
C2—C3	1.394 (3)	C10—Cl1	1.745 (2)
C3—C4	1.384 (3)	C11—C12	1.378 (3)
C3—H3	0.9500	C11—H11	0.9500
C4—C5	1.386 (3)	C12—C13	1.381 (3)
C4—H4	0.9500	C12—H12	0.9500
C5—C6	1.378 (3)	C13—H13	0.9500
C5—H5	0.9500	C6—H6	0.9500
C7—C6	1.387 (3)		
			
C1—N1—C8	127.52 (19)	C9—C8—C13	119.9 (2)
C1—N1—H1	117.3 (16)	C9—C8—N1	122.8 (2)
C8—N1—H1	114.8 (16)	C13—C8—N1	117.3 (2)
O1—C1—N1	122.8 (2)	C10—C9—C8	118.1 (2)
O1—C1—C2	121.1 (2)	C10—C9—H9	121.0
N1—C1—C2	116.09 (19)	C8—C9—H9	121.0
C7—C2—C3	119.1 (2)	C11—C10—C9	122.9 (2)
C7—C2—C1	123.4 (2)	C11—C10—Cl1	119.38 (18)
C3—C2—C1	117.5 (2)	C9—C10—Cl1	117.77 (18)
C4—C3—C2	120.1 (2)	C12—C11—C10	118.1 (2)
C4—C3—H3	119.9	C12—C11—H11	120.9
C2—C3—H3	119.9	C10—C11—H11	120.9
C3—C4—C5	120.5 (2)	C11—C12—C13	121.0 (2)
C3—C4—H4	119.8	C11—C12—H12	119.5
C5—C4—H4	119.8	C13—C12—H12	119.5
C6—C5—C4	119.6 (2)	C12—C13—C8	120.1 (2)
C6—C5—H5	120.2	C12—C13—H13	120.0
C4—C5—H5	120.2	C8—C13—H13	120.0
C6—C7—C2	120.2 (2)	C5—C6—C7	120.4 (2)
C6—C7—H7	119.9	C5—C6—H6	119.8
C2—C7—H7	119.9	C7—C6—H6	119.8

### Hydrogen-bond geometry (Å, °)

**Table:**

*D*—H···*A*	*D*—H	H···*A*	*D*···*A*	*D*—H···*A*
N1—H1···O1^i^	0.88 (2)	1.99 (2)	2.841 (2)	163 (2)
C7—H7···O1^i^	0.95	2.45	3.228 (3)	139
C13—H13···O1^i^	0.95	2.71	3.301 (3)	121

Symmetry codes: (i) *x*, −*y*+1/2, *z*−1/2.

## References

[bb1] Allen, F. H., Johnson, O., Shields, G. P., Smith, B. R. & Towler, M. (2004). *J. Appl. Cryst.* **37**, 335–338.

[bb2] Allen, F. H., Kennard, O., Watson, D. G., Brammer, L., Orpen, A. G. & Taylor, R. (1987). *J. Chem. Soc. Perkin Trans. 2*, pp. S1–19.

[bb3] Bernstein, J., Davis, R. E., Shimoni, L. & Chang, N.-L. (1995). *Angew. Chem. Int. Ed. Engl.* **34**, 1555–1573.

[bb4] Bruker (2006). *APEX2*, *SAINT* and *SADABS* Bruker AXS Inc., Madison, Wisconsin, USA.

[bb5] Gowda, B. T., Foro, S., Sowmya, B. P. & Fuess, H. (2008). *Acta Cryst.* E**64**, o1243. 10.1107/S1600536808017017 PMC2961786 21202878

[bb6] Gowda, B. T., Sowmya, B. P., Kožíšek, J., Tokarčík, M. & Fuess, H. (2007*a*). *Acta Cryst.* E**63**, o2906. 10.1107/S1600536807066937 PMC2915381 21200902

[bb7] Gowda, B. T., Sowmya, B. P., Tokarčík, M., Kožíšek, J. & Fuess, H. (2007*b*). *Acta Cryst.* E**63**, o3326. 10.1107/S1600536807066937 PMC2915381 21200902

[bb8] Gowda, B. T., Sowmya, B. P., Tokarčík, M., Kožíšek, J. & Fuess, H. (2007*c*). *Acta Cryst.* E**63**, o3365. 10.1107/S1600536807066937 PMC2915381 21200902

[bb9] Gowda, B. T., Tokarčík, M., Kožíšek, J., Sowmya, B. P. & Fuess, H. (2008). *Acta Cryst.* E**64**, o462. 10.1107/S1600536808001311 PMC2960316 21201488

[bb10] Hunter, K. A. & Simpson, J. (1999). *TITAN2000* University of Otago, New Zealand.

[bb11] Macrae, C. F., Bruno, I. J., Chisholm, J. A., Edgington, P. R., McCabe, P., Pidcock, E., Rodriguez-Monge, L., Taylor, R., van de Streek, J. & Wood, P. A. (2008). *J. Appl. Cryst.* **41**, 466–470.

[bb12] Saeed, A., Khera, R. A. & Simpson, J. (2010). *Acta Cryst.* E**66**, o911–o912. 10.1107/S1600536810010378 PMC2983768 21580722

[bb13] Sheldrick, G. M. (2008). *Acta Cryst.* A**64**, 112–122. 10.1107/S0108767307043930 18156677

[bb14] Spek, A. L. (2009). *Acta Cryst.* D**65**, 148–155. 10.1107/S090744490804362X PMC2631630 19171970

[bb15] Westrip, S. P. (2010). *J. Appl. Cryst.* **43**, 920–925.

